# Frailty and the prediction of dependence and mortality in low- and middle-income countries: a 10/66 population-based cohort study

**DOI:** 10.1186/s12916-015-0378-4

**Published:** 2015-06-10

**Authors:** Jotheeswaran AT, Renata Bryce, Matthew Prina, Daisy Acosta, Cleusa P Ferri, Mariella Guerra, Yueqin Huang, Juan J. Llibre Rodriguez, Aquiles Salas, Ana Luisa Sosa, Joseph D. Williams, Michael E. Dewey, Isaac Acosta, Zhaorui Liu, John Beard, Martin Prince

**Affiliations:** Centre for Global Mental Health, Health Service and Population Research Department, Institute of Psychiatry, King’s College, De Crespigny Park, London, SE5 8AF UK; Universidad Nacional Pedro Henriquez Ureña (UNPHU), Internal Medicine Department, Geriatric Section, Km 7 1/2, Av. John F. Kennedy, Santo Domingo, 1423 Dominican Republic; Universidade Federal de Sao Paulo, Department of Psychobiology, Rua Napoleão de Barros, 925, 3rd floor, São Paulo, SP, 04024-002 Brazil; Institute of Education and Health Sciences at the Hospital Alemao Oswaldo Cruz, Rua João Juliao, 245, First floor, Sao Paulo, SP, 01323-903 Brazil; Instituto de la Memoria y Desordenes Relacionados, Av. Constructores 1230, La Molina, Lima Perú; Peking University, Institute of Mental Health, 51 Huayuan N Rd, Haidian, Beijing 100083 China; Facultad de Medicina Finley-Albarran, Medical University of Havana, Calle 146 No. 2504, Playa municipality, Havana, Cuba; Medicine Department, Caracas University Hospital; Faculty of Medicine, Universidad Central de Venezuela, Edif. Decanato de Medicina, Los Chaguaramos, Caracas-9995 Venezuela; National Institute of Neurology and Neurosurgery of Mexico, Autonomous National University of Mexico, Delegacion Tlalpan, Coyoacán -, 4510 Mexico; Voluntary Health Services, Taramani, Chennai- 6000113 India; Indian Institute of Public Health Hyderabad, Public Health Foundation of India, Plot no 1, ANV Arcade, Amar Co-operative society, Kavuri Hills, Madhapur, Hyderabad -, 500033, Telangana India; Department of Ageing and Life Course, World Health Organization, Avenue Appia 201211, Geneva 27, Switzerland

**Keywords:** Aged, Frailty, Developing countries, Disability, Geriatric assessment, Epidemiology, Long-term care, Mortality

## Abstract

**Background:**

In countries with high incomes, frailty indicators predict adverse outcomes in older people, despite a lack of consensus on definition or measurement. We tested the predictive validity of physical and multidimensional frailty phenotypes in settings in Latin America, India, and China.

**Methods:**

Population-based cohort studies were conducted in catchment area sites in Cuba, Dominican Republic, Venezuela, Mexico, Peru, India, and China. Seven frailty indicators, namely gait speed, self-reported exhaustion, weight loss, low energy expenditure, undernutrition, cognitive, and sensory impairment were assessed to estimate frailty phenotypes. Mortality and onset of dependence were ascertained after a median of 3.9 years.

**Results:**

Overall, 13,924 older people were assessed at baseline, with 47,438 person-years follow-up for mortality and 30,689 for dependence. Both frailty phenotypes predicted the onset of dependence and mortality, even adjusting for chronic diseases and disability, with little heterogeneity of effect among sites. However, population attributable fractions (PAF) summarising etiologic force were highest for the aggregate effect of the individual indicators, as opposed to either the number of indicators or the dichotomised frailty phenotypes. The aggregate of all seven indicators provided the best overall prediction (weighted mean PAF 41.8 % for dependence and 38.3 % for mortality). While weight loss, underactivity, slow walking speed, and cognitive impairment predicted both outcomes, whereas undernutrition predicted only mortality and sensory impairment only dependence. Exhaustion predicted neither outcome.

**Conclusions:**

Simply assessed frailty indicators identify older people at risk of dependence and mortality, beyond information provided by chronic disease diagnoses and disability. Frailty is likely to be multidimensional. A better understanding of the construct and pathways to adverse outcomes could inform multidimensional assessment and intervention to prevent or manage dependence in frail older people, with potential to add life to years, and years to life.

## Background

Most definitions of frailty share two core features; firstly, an underlying progressive age-related decline in physiologic systems, with large individual variation, and second, a consequent decreased functional reserve capacity, conferring vulnerability for failure in the face of environmental stressors [1]. Sustained interest in the construct stems mainly from its predictive validity, confirmed through increased risks of adverse health and social outcomes for older people – morbidity, hospitalization, falls and fractures, disability, dependence, institutionalization and death [2–5]. The process of becoming frail may be delayed, slowed, or even partly reversed by interventions targeted early in the process of functional decline [6]. Trials of complex interventions, designed to promote independence in moderately frail older people have shown potential benefits. These are important findings with global implications [7–9]. Population ageing is proceeding apace in all world regions, but the populations of many low-, and particularly middle-income countries are ageing more rapidly than any country in the past; two-thirds of the world’s older people live in low- and middle-income countries (LMICs), rising to 80 % by 2050 [10]. While morbidities mediate the relationship between population ageing and societal costs, the relationships with chronological age are variable, and potentially amenable to influence from public health, health, and social care interventions [11].

A clearer understanding of the nature of the frailty construct, and its relations to adverse outcomes is needed to inform and prioritise intervention strategies. Dissatisfaction has been expressed with current models of frailty and approaches to measurement, with at least seventeen different conceptual definitions proposed [12–14]. As originally defined by Fried et al.[4], frailty was a unidimensional, largely physical construct identified by the presence of three or more of five indicators – exhaustion, weight loss, weak grip strength, slow walking speed, and low energy expenditure. Others have proposed widening the scope to include, for example, cognitive or sensory domains [15, 16]. Incorporating diseases and disability has been particularly controversial [3]; if frailty represents an underlying vulnerability, then disease and disability may be among the predicted outcomes rather than part of the construct itself. ‘Frailty indices’ neglect this distinction, assessing age-dependent accumulation of a wide range of health indicators; symptoms, signs, conditions, diseases, and disabilities [17, 18].

In summary, it is unclear whether frailty is best considered a unidimensional or multidimensional construct. Its boundaries remain unclear, with tentative evidence to support the inclusion of cognitive ageing as a relevant aspect of frailty. We therefore set out to test, empirically, the utility of two widely applied frailty constructs, the physical frailty phenotype, as originally conceptualised by Fried et al.[4], and a broader, multidimensional frailty model based on deficiencies in physical, nutritive, cognitive, and sensory domains of functioning [16, 17]. We had three questions. Are older people defined as frail according to these paradigms at higher risk of dependence and death, even after controlling for major chronic diseases and disability? Does the aggregate of the individual indicators provide a better prediction of these outcomes (judged by population attributable fraction (PAF) derived from multivariable models) than the dichotomised or ordinal frailty scores? Are different frailty indicators differentially associated with the incidence of dependence and mortality? We addressed these questions in a large population-based cohort study in seven LMICs, in which settings little previous research into frailty has been conducted.

## Method

### Settings and study design

The 10/66 Dementia Research Group’s (10/66 DRG) population-based studies of ageing and dementia in LMICs comprised baseline surveys of all older people aged 65 years and over living in geographically-defined catchment areas in seven countries, with a follow-up 3–5 years later. For the current analyses, this comprises urban and rural sites in China, Mexico and Peru, and urban sites in Cuba, Dominican Republic, Venezuela and India. Baseline population-based surveys were carried out between 2003 and 2007, and incidence wave follow-up assessments between 2008 and 2010. For India, the follow-up comprised a mortality sweep only. The design of the baseline and follow-up phases of the 10/66 DRG research program have been described in detail elsewhere [19]. Here, we will describe aspects directly relevant to the analyses presented in this paper.

### Ethical issues

Participants were recruited following informed signed consent. Persons with dementia who lacked capacity for consent were recruited on the basis of a relative’s signed agreement. Illiterate persons were read the information sheet and consent form, and invited to express their consent verbally, which was witnessed. Studies were approved by local ethical committees as well as by the ethical committee of the Institute of Psychiatry, King’s College London.

### Exposures – Frailty

#### Frailty indicators

We assessed seven indicators of frailty, namely exhaustion, weight loss, slow walking speed, low energy expenditure (physical inactivity), undernutrition, and cognitive and sensory impairment. These were operationalized as follows:

1. Exhaustion: assessed using a single item (Q.48.1) from the Geriatric Mental Status examination. Participants who reported feeling worn out or exhausted were considered to have this frailty [20].
2. Weight loss: Self-reported weight loss was assessed using a single item from the Geriatric Mental State (Q53.1) “Have you lost any weight in the last three months?” Those reporting weight loss of 10 lbs (4.5 kg) or more in last three months were considered to have this frailty [20].
3. Slow walking speed: assessed using a standard timed walking test in which the participant was asked to walk 5 metres at usual speed, turn, and return to the starting point. Those taking 16 seconds or longer to complete the task were considered to have a slow walking speed.
4. Low energy expenditure: in response to the question “Taking into account both work and leisure, would you say that you are: very, fairly, not very or not at all physically active?” Those that rated themselves not at all physical active were considered physically inactive.
5. Undernutrition: assessed through the measurement of mid-upper arm circumference, those with a circumference of <22 cm were considered to be frail. This cut-point is used in the Mini Nutritional Assessment® to identify the most severe level of undernutrition according to this index [21].
6. Cognitive impairment: cognitive function was assessed using the Community Screening Instrument for Dementia COGSCORE, which tests multiple domains of cognitive function, and has been found to have robust cross-cultural measurement properties in the 10/66 study sites [22]. Frailty was defined according to the higher of two possible cut-points (29.5, for ‘possible dementia’) in order to identify cognitive impairment beyond dementia.
7. Sensory impairment: assessed according to self-report (from two separate items) of having ‘eyesight problems’ or ‘hearing problems or deafness’, which interfered with activities to at least some extent.

### Frailty phenotypes

#### Physical frailty model

Fried et al.’s physical frailty model [23] proposes five specific and measurable indicators to identify frailty (exhaustion, weight loss, weak grip strength, slow walking speed, and low energy expenditure). Individuals are identified as frail if they meet three or more of the five criteria, as intermediate if they meet one or two, and as non-frail if they meet none of the five criteria [4]. We applied our exhaustion, weight loss, slow walking speed, and low energy expenditure indicators. Since handgrip strength was not measured we considered participants as frail if they fulfilled two or more of the four frailty indicators.

#### Multidimensional frailty

The approach developed in the Alameda County study comprised 16 self-reported items grouped into four domains of functioning (physical, nutrition, cognitive, and sensory) [16]. The physical functioning domain included dizziness, loss of balance, weakness in the arms, and weakness in the legs. The nutritive functioning domain included loss of appetite and unexplained weight loss. The cognitive functioning domain included memory and attention difficulties. The sensory functioning domain included vision and hearing difficulties in different situations. Participants were classified as frail if they had difficulties in two or more domains. We applied our slow walking speed, undernutrition, cognitive impairment, and sensory impairment indicators.

### Covariates – measures of socio-demographic circumstances, morbidity, and disability

Age, sex, and educational level were important determinants of mortality [24] and dependence in our LMIC sites [25]. Participants’ ages were established during the baseline interview, from stated age, official documentation, informant report, and, in the case of discrepancy, age according to an event calendar. We also recorded the participant’s gender and educational level (none; some but did not complete primary; completed primary; completed secondary; tertiary).

We summarised the impact of physical, mental and cognitive health through measurement and control for stroke, physical impairments, dementia and depression – conditions previously shown to make a substantial contribution to disability and dependence [25, 26]. These were assessed as follows:

1. Dementia diagnosed according to the cross-culturally developed, calibrated and validated 10/66 dementia diagnosis algorithm, on the basis of cognitive testing, clinical mental state interview and informant interview [22].
2. Self-reported stroke, confirmed by the interviewer as having characteristic symptoms lasting for more than 24 hours [27].
3. Number of self-reported limiting physical impairments from a list of nine (arthritis or rheumatism; persistent cough; breathlessness, difficulty breathing or asthma; high blood pressure; heart trouble or angina; stomach or intestine problems; faints or blackouts; paralysis, weakness or loss of one leg or arm; skin disorders such as pressure sores, leg ulcers or severe burns).
4. International Classification of Diseases-10 depressive episode (mild, moderate or severe), derived using a computerised algorithm applied to a structured clinical interview, the Geriatric Mental State [26].

Disability was assessed as activity limitation and participation restriction measured by the World Health Organization Disability Assessment Scale 2.0, developed as a culture-fair assessment tool for use in cross-cultural comparative epidemiological and health services research [28]. We had previously demonstrated measurement invariance across the sites included in our survey.

### Outcomes

In the incidence wave we sought to trace and re-interview all baseline survey participants. We first called on their residence at baseline, revisiting on up to four occasions. Where the participant was no longer resident we sought information regarding their vital status (if known) and/or current residence, assisted by having recorded, at baseline, the names and addresses of three non-coresident friends or family members. Where participants had moved away, we sought to re-interview them, even if they had moved out of the original catchment area, by telephone if necessary. Where a participant had died, we recorded date of death, and completed a verbal autopsy interview with a suitable key informant.

Dependence (need for care) was identified through a series of open-ended questions to a key informant: Who shares the home? What kind of help does the participant need inside and outside of the home? Who, in the family, is available to care? What help do you provide? Do you help to organise care? Is there anyone else in the family who is more involved in helping? What do they do? What about friends and neighbours, what do they do? The interviewer then coded whether the participant required no care, care some of the time, or care much of the time [29]. The same approach was used at baseline and follow-up surveys. Those with no needs for care at baseline were considered to be at risk for the incidence of dependence, and those among them who were rated as needing care some of the time or much of the time at follow-up were considered to have incident dependence.

### Analysis

All data was double entered into EPIDATA software and data analysis was performed using STATA version 10. We describe the principal characteristics of the mortality cohort (the whole baseline survey sample, at risk for mortality), and the dependence cohort (those with no needs for care at baseline, hence at risk for the onset of dependence). Person-years risk for the onset of dependence was calculated as the interval between baseline and follow-up assessment, or the mid-point of this interval for those who developed dependence. We used Poisson regression to estimate incidence rate ratios (IRR) for associations with incident dependence. We used Cox’s proportional hazards regression to estimate hazard ratios for associations with mortality. Survival times were censored on the date of death, or the date of follow-up for those who were re-interviewed, or the median date of follow-up interview in that site for those refusing interview. We first assessed the associations of the dichotomized frailty syndromes (defined according to physical and multidimensional frailty criteria) with both outcomes, controlling incrementally for age, sex and education (model 1), these factors plus health conditions (dementia, depression, number of physical impairments and stroke – model 2), and all of these factors plus disability (model 3). We ran the models in each site, and then used a fixed or random effects meta-analysis to combine them. Higgins I^2^ was computed, estimating the proportion of between-site variability in the estimates accounted for by heterogeneity, as opposed to sampling error; up to 40 % heterogeneity is conventionally considered negligible, while up to 60 % may reflect moderate heterogeneity [30]. For model 2 (controlling for age, sex, education and health conditions, but not disability) we used the STATA aflogit command to calculate PAF % with 95 % confidence intervals (CIs) for the contribution of frailty syndromes to the incidence of dependence and mortality, comparing the dichotomised frailty syndrome with two alternative approaches; either using the number of indicators (0 to 4) as an ordinal scale, or the aggregate effect of the four individual indicators. We also estimated the aggregate effect of all seven frailty indicators entered simultaneously. The STATA aflogit command estimates the individual and combined attributable fractions robustly from within the Poisson regression framework. PAFs represent the proportion of the incidence of the outcome that could theoretically be avoided if the exposure could be removed from the population, assuming causal relationships estimated free of confounding. Finally, we estimated and compared the effects of each of the seven individual frailty indicators for associations with incident dependence (pooled meta-analysed IRR) and mortality (pooled meta-analysed HR) controlling as per model 2 above for demographic variables and health conditions.

## Results

The mortality cohort comprised 13,924 individuals at baseline. Vital status was ascertained at follow-up in 88.9 % (n = 12,373) ranging from 74.4 % to 100 % by site. Median years of follow-up ranged from 2.8 to 5.0 years, because of the variation among sites in the period in which the baseline surveys were conducted; overall, 47,439 person-years of mortality follow-up were accumulated. Mortality rates ranged from 27.3/1000 person-years (urban Peru) to 70.0/1000 person-years in urban India. The dependence cohort comprised 11,251 individuals, with no needs for care at baseline; 7,910 (70.3 %) were successfully reinterviewed (64.6–77.4 % by site). Deaths accounted for 1,510 (13.4 %), 724 (6.4 %) refused, and 1,116 (9.9 %) could not be contacted. The incidence of dependence ranged from 22.3/1000 (rural China) to 50.0/1000 person-years (urban China). In the full baseline sample (mortality cohort) the prevalence of frailty was 17.5 % according to the physical frailty and 29.1 % according to multidimensional frailty criteria. There was considerable variation among sites, with the highest prevalence observed in the Dominican Republic (34.6 % physical and 47.8 % multidimensional frailty) and the lowest in urban China (7.8 % physical and 11.3 % multidimensional frailty). Prevalence of frailty according to multidimensional criteria was generally higher than that for physical frailty. Among those without needs for care at baseline (the dependence cohort), the prevalence of frailty was somewhat lower, 13.5 % according to physical frailty criteria, and 22.5 % according to multidimensional criteria (Table 1).

**Table 1:** Cohort characteristics

	Cuba	Dominican Republic	Peru (urban)	Peru (rural)	Venezuela	Mexico (urban)	Mexico (rural)	China (urban)	China (rural)	India (urban)	All centres combined
**MORTALITY COHORT**											
Baseline sample (alive at baseline)	2813	2011	1381	552	1997	1003	1000	1160	1002	1005	13924
Vital status determined (% of baseline sample)	2637 (93.7 %)	1706 (84.8 %)	1245 (90.2 %)	507 (91.8 %)	1697 (84.5 %)	909 (90.6 %)	933 (93.3 %)	989 (85.2 %)	1002 (100.0 %)	748 (74.4 %)	12373 (88.9 %)
Deaths (% of those with vital status determined)	609 (23.1 %)	467 (27.4 %)	98 (7.9 %)	54 (10.6 %)	200 (11.8 %)	99 (10.9 %)	110 (11.8 %)	224 (22.6 %)	291 (29.0 %)	154 (20.6 %)	2306 (18.6 %)
Person years of follow-up	10852.5	7448.6	3592.7	1764.1	7031.1	2667.1	2689.3	4630.6	4563.3	2198.7	47437.9
Mortality rate (per 1000 person-years)	56.1 (51.8–60.7)	62.7 (57.2–68.6)	27.3 (22.3 –33.1)	30.6 (23.2–39.6)	28.4 (24.7–32.6)	37.1 (30.3–45.0)	40.9 (33.8–49.1)	48.4 (42.3–55.0)	63.8 (56.8–71.4)	70.0 (59.6–81.8)	56.1 (51.8–60.7)
Median years of follow-up (25^th^ and 75^th^ centile)	4.2 (3.5–5.0)	5.0 (3.6–5.1)	2.8 (2.4–3.4)	3.7 (3.6–3.8)	4.2 (4.0–4.8)	3.0 (2.9–3.2)	3.0 (2.9–3.1)	4.9 (4.6–5.3)	4.9 (4.4–5.2)	2.9 (2.5–3.6)	3.9 (3.0–4.9)
Mean age at baseline (SD)	75.2 (7.1)	75.4 (7.6)	75.0 (7.4)	74.1 (7.3)	72.3 (6.8)	74.4 (6.6)	74.1 (6.6)	74.1 (6.3)	72.4 (6.0)	71.4 (6.1)	74.1 (7.0)
Female sex (%)	1714 (65.0 %)	1130 (66.3 %)	805 (64.7 %)	270 (53.2 %)	1072 (63.2 %)	605 (66.5 %)	569 (60.9 %)	560 (56.6 %)	556 (55.5 %)	422 (57.2 %)	7703 (62.3 %)
Did not complete primary education (%)	661 (25.1 %)	1211 (71.7 %)	114 (9.2 %)	206 (41.3 %)	499 (30.0 %)	530 (58.4 %)	787 (84.2 %)	346 (35.0 %)	693 (69.2 %)	492 (66.0 %)	5539 (45.1 %)
Fried frailty model	554 (21.0 %)	591 (34.6 %)	323 (25.9 %)	87 (17.2 %)	187 (11.0 %)	92 (10.1 %)	79 (8.5 %)	77 (7.8 %)	87 (8.7 %)	85 (11.4 %)	2162 (17.5 %)
Multidimensional frailty model	889 (33.7 %)	816 (47.8 %)	351 (28.2 %)	130 (25.6 %)	340 (20.0 %)	208 (22.9 %)	338 (36.2 %)	112 (11.3 %)	225 (22.5 %)	195 (26.1 %)	3604 (29.1 %)
**DEPENDENCE COHORT**											
Baseline sample (no needs for care at baseline)	2225	1770	1246	524	1754	889	918	977	948		11251
Re-interviewed (% of baseline sample)	1662 (74.7 %)	1144 (64.6 %)	830 (66.6 %)	399 (76.1 %)	1154 (65.8 %)	688 (77.4 %)	664 (72.3 %)	671 (68.7 %)	698 (73.6 %)	–	7910 (70.3 %)
Incident dependence (% of those re-interviewed)	233 (14.0 %)	242 (21.2 %)	95 (11.4 %)	38 (9.5 %)	181 (15.7 %)	90 (13.1 %)	90 (13.6 %)	151 (22.5 %)	74 (10.6 %)	–	1194 (15.1 %)
Person years of follow-up	7031.6	5002.0	2317.1	1414.5	4702.4	1979.3	1900.4	3020.7	3320.7	–	30688.8
Incidence rate (per 1000 person-years)	33.1 (29.1–37.6)	48.4 (42.6–54.8)	41.0 (33.4–49.9)	26.9 (19.3–36.5)	38.5 (33.2–44.4)	45.5 (36.8–55.6)	47.4 (38.3–57.9)	50.0 (42.5–58.5)	22.3 (17.6–27.8)	–	38.9 (36.7–41.2)
Median years of follow-up (25^th^ and 75^th^ centile)	4.3 (3.6–5.1)	5.0 (4.8–5.2)	2.8 (2.4–3.2)	3.7 (3.6–3.7)	4.2 (4.0–4.7)	3.0 (2.9–3.2)	3.0 (2.9–3.1)	4.9 (4.4–5.3)	4.9 (4.7–5.3)	–	4.0 (3.0–4.9)
Mean age at baseline (SD)	73.5 (6.2)	73.6 (6.6)	74.1 (6.8)	73.2 (6.7)	71.1 (5.8)	73.4 (6.0)	73.5 (6.3)	72.4 (5.3)	71.0 (5.1)	–	72.9 (6.2)
Female sex (%)	1074 (64.6 %)	784 (68.7 %)	545 (65.7 %)	213 (53.4 %)	742 (64.3 %)	453 (65.8 %)	412 (62.0 %)	395 (58.9 %)	397 (56.9 %)	–	5015 (63.4 %)
Did not complete primary education (%)	356 (21.4 %)	797 (69.9 %)	69 (8.4 %)	153 (38.9 %)	302 (26.3 %)	379 (55.3 %)	549 (82.6 %)	226 (33.7 %)	467 (66.9 %)	–	3298 (41.8 %)
Fried frailty model	258 (15.5 %)	347 (30.3 %)	185 (22.3 %)	58 (14.5 %)	89 (7.7 %)	57 (8.3 %)	37 (5.6 %)	5 (0.7 %)	33 (4.7 %)	–	1069 (13.5 %)
Multidimensional frailty model	397 (23.9 %)	457 (39.9 %)	181 (21.8 %)	84 (21.1 %)	182 (15.8 %)	121 (17.6 %)	221 (33.3 %)	23 (3.4 %)	115 (16.5 %)	–	1781 (22.5 %)

The meta-analysed effects of frailty on the incidence of dependence and mortality are presented in Table 2. Both the physical and the multidimensional dichotomous frailty definitions independently predicted the onset of dependence and mortality. Effect sizes were progressively attenuated after controlling sequentially for demographic factors, chronic health conditions and disability, but remained statistically significant. The heterogeneity among sites in the estimates of association is minimal to moderate throughout, and only those for the association between frailty according to the multidimensional criteria and mortality are statistically significant.

**Table 2:** Meta-analysed effects of dichotomous frailty indicators (physical and multidimensional frailty models) on the incidence of dependence and mortality, controlling sequentially for health conditions and disability

	Model 1 (age, sex and education)	Model 2 (model 1 + health conditions)^a^	Model 3 (model 2 + disability)^b^
**Physical frailty phenotype**			
Dependence (IRR)^c^	F^d^ = 1.77 (1.53–2.04)	F = 1.43 (1.24–1.64)	F = 1.28 (1.10–1.48)
Heterogeneity	Cochrane’s Q 14.1, 8 df, *P* = 0.08	Cochrane’s Q 13.9, 8 df, *P* = 0.09	Cochrane’s Q 10.0, 8 df, *P* = 0.27
	Higgins I^2^ = 43 (0–74)	Higgins I^2^ = 42 (0–73)	Higgins I^2^ = 20 (0–61)
Mortality (HR)^e^	F = 1.89 (1.72–2.08)	F = 1.51 (1.36–1.68)	F = 1.18 (1.06–1.33)
	R^f^ = 1.97 (1.68–2.31)		
Heterogeneity	Cochrane’s Q 20.1, 9 df, *P* = 0.02	Cochrane’s Q 15.0, 9 df, *P* = 0.09	Cochrane’s Q 10.8, 9 df, *P* = 0.29
	Higgins I^2^ = 55 (9–78)	Higgins I^2^ = 40 (0–71)	Higgins I^2^ = 16 (0–58)
**Multidimensional phenotype**			
Dependence (IRR)	F = 2.15 (1.88–2.46)	F = 1.46 (1.27–1.68)	F = 1.36 (1.18–1.57)
Heterogeneity	Cochrane’s Q 2.9, 8 df, *P* = 0.95	Cochrane’s Q 6.1, 8 df, *P* = 0.64	Cochrane’s Q 3.5, 8 df, *P* = 0.90
	Higgins I^2^ = 0 (0–65)	Higgins I^2^ = 0 (0–65)	Higgins I^2^ = 0 (0–65)
Mortality (HR)	F = 1.96 (1.78–2.15)	F = 1.54 (1.39–1.71)	F = 1.38 (1.24–1.54)
	R = 1.94 (1.66–2.28)	R = 1.53 (1.29–1.81)	R = 1.36 (1.14–1.62)
Heterogeneity	Cochrane’s Q 21.1, 9 df, *P* = 0.01	Cochrane’s Q 19.7, 9 df, *P* = 0.02	Cochrane’s Q 19.8, 9 df, *P* = 0.02
	Higgins I^2^ = 57 (14–79)	Higgins I^2^ = 54 (7–78)	Higgins I^2^ = 55 (8–78)

^a^10/66 or DSM-IV dementia diagnosis, ICD-10 depression, number of physical impairments and stroke; ^b^WHO Disability Assessment Scale 2.0; ^c^IRR, Incidence rate ratio; ^d^F, Pooled fixed effect; ^e^HR, Hazard ratio; ^f^R, Pooled random effect (estimated only in the presence of statistically significant heterogeneity)

Next, we compared the physical and multidimensional frailty phenotypes as dichotomised syndromes, ordinal scales, and as the aggregate of their individual indicators, with respect to the PAFs for their independent contribution to the onset of dependence (Table 3) and mortality (Table 4). For both outcomes, the contributions of the ordinal scale and of the aggregate of the individual indicators of frailty consistently exceeded those for the dichotomous definition, and the aggregate contribution of the individual indicators generally exceeded that of the ordinal scale. For the physical frailty models, the PAFs for dependence for the dichotomous definition range from 3.1 % to 26.7 % (weighted mean 9.5 %), for the ordinal scale from 3.3 % to 43.4 % (weighted mean 18.6 %), and for the individual indicators from 3.6 % to 62.1 % (weighted mean 23.2 %). For the multidimensional frailty model, the PAFs for dependence for the dichotomous definition range from 7.0 % to 31.0 % (weighted mean 18.0 %), for the ordinal scale from 5.5 % to 47.7 % (weighted mean 31.3 %), and for the individual indicators from 15.2 % to 58.3 % (weighted mean 36.9 %). The PAFs for mortality for the dichotomous definition of the Fried frailty model range from 0.8 % to 18.9 % (weighted mean 10.5 %), for the ordinal scale from 0.6 % to 40.3 % (weighted mean 20.9 %), and for the individual indicators from 8.9 % to 46.5 % (weighted mean 25.1 %). For the multidimensional frailty model, the PAF for dependence for the dichotomous definition range from 5.3 % to 42.2 % (weighted mean 19.6 %), for the ordinal scale from 4.3 % to 49.8 % (weighted mean 28.3 %), and for the individual indicators from 7.7 % to 56.2 % (weighted mean 33.4 %). In general, the aggregate effect of all seven indicators exceeded that for any of the unidimensional or multidimensional operationalisations with a weighted mean PAF of 41.8 % for dependence and 38.3 % for mortality.

**Table 3:** Population attributable fractions (PAF % with 95 % confidence intervals) for the independent contribution of frailty to the incidence of dependence, when operationalised as dichotomous categories, ordinal scales or individual indicators

	Physical frailty criteria			Multidimensional frailty criteria			Individual indicators from both frailty paradigms
	Dichotomous	Ordinal scale	Individual indicators	Dichotomous	Ordinal scale	Individual indicators	
Cuba	10.3 (2.9–17.2)	27.0 (15.2–37.2)	28.0 (16.4–38.1)	23.5 (10.7–34.5)	40.9 (26.7–52.3)	43.5 (30.7–54.0)	44.5 (31.8-54.9)
Dominican Republic	3.1 (0.0–8.6)	3.3 (0.0–15.3)	3.6 (0.0–8.3)	7.6 (0.0–18.4)	18.5 (0.0–34.9)	22.1 (1.7–38.2)	24.4 (4.1-40.4)
Peru (urban)	9.4 (0.0–20.1)	11.1 (0.0–29.2)	17.8 (0.0–39.4)	31.0 (4.2–50.3)	46.6 (15.6–66.3)	58.3 (37.6–72.1)	60.7 (43.2-72.8)
Peru (rural)	26.7 (2.3–45.2)	43.4 (0.0–68.8)	62.1 (0.0–86.7)	22.9 (0.0–48.5)	47.7 (0.0–73.7)	49.9 (1.4–74.5)	72.5 (1.5-92.3)
Venezuela	15.5 (3.9–25.7)	30.8 (12.7–45.1)	30.4 (10.1–46.1)	21.5 (6.1–34.4)	47.6 (30.5–60.5)	49.0 (30.8–62.4)	54.6 (35.7-68.0)
Mexico (urban)	5.9 (0.0–18.5)	16.2 (0.0–40.0)	24.8 (0.0–49.9)	17.0 (0.0–35.2)	31.6 (1.2–52.6)	42.0 (9.0–63.1)	42.8 (7.8-64.5)
Mexico (rural)	11.1 (0.0–23.3)	13.0 (0.0–32.7)	27.0 (0.0–47.0)	7.0 (0.0–25.0)	5.5 (0.0–36.0)	15.2 (0.0–35.5)	25.6 (0.0-46.7)
China (urban)	10.5 (0.0–33.2)	24.6 (0.0–46.4)	26.1 (0.0–47.5)	14.4 (0.0–34.9)	24.0 (0.0–46.4)	28.0 (2.7–46.6)	38.5 (11.4-57.3)
China (rural)	Inverse association	Inverse association	8.1 (0.0–43.8)	13.0 (0.0–37.6)	5.7 (0.0–43.3)	16.6 (0.0–38.3)	20.4 (0.0-58.7)
Weighted mean	9.5	18.6	23.2	18.0	31.3	36.9	41.8

**Table 4:** Population attributable fractions (PAF % with 95 % confidence intervals) for the independent contribution of frailty to the incidence of mortality, when operationalised as dichotomous categories, ordinal scales or individual indicators

	Physical frailty criteria			Multidimensional criteria			Individual indicators from both frailty paradigms
	Dichotomous	Ordinal scale	Individual indicators	Dichotomous	Ordinal scale	Individual indicators	
Cuba	8.8 (6.5–11.0)	16.5 (12.0–20.8)	13.6 (8.1–18.7)	20.1 (15.5–24.4)	30.2 (24.3–35.6)	30.4 (24.0–36.2)	29.8 (22.8–36.1)
Dominican Republic	5.4 (2.3–7.7)	17.8 (12.4–22.8)	20.5 (14.9–25.7)	13.1 (8.6–17.5)	21.8 (14.4–28.6)	30.0 (23.4–36.0)	33.5 (27.2–39.3)
Peru (urban)	13.6 (4.9–21.6)	26.0 (11.9–37.9)	40.0 (22.2–53.8)	42.2 (22.5–56.9)	49.8 (28.7–64.6)	56.2 (38.9–68.6)	56.7 (39.5–69.0)
Peru (rural)	0.8 (0.0–10.1)	0.6 (0.0–19.7)	13.6 (0.0–30.1)	27.5 (5.5–44.4)	29.2 (0.0–52.7)	34.5 (7.1–53.9)	34.9 (3.8–55.9)
Venezuela	17.5 (11.2–23.4)	32.5 (23.0–40.8)	38.1 (24.5–49.3)	21.9 (12.4–30.4)	41.8 (29.3–52.1)	51.6 (37.8–62.3)	55.0 (41.3–65.6)
Mexico (urban)	12.8 (2.9–21.7)	20.3 (0.0–36.8)	8.9 (0.2–16.8)	15.7 (0.7–28.4)	29.2 (4.1–47.7)	22.3 (0.0–44.9)	24.1 (1.8–41.4)
Mexico (rural)	18.9 (9.4–27.4)	40.3 (25.8–52.0)	46.5 (32.3–57.8)	10.9 (0.0–23.6)	16.4 (0.0–33.9)	36.3 (17.3–50.9)	49.0 (35.1–59.9)
China (urban)	14.5 (10.4–18.4)	30.7 (24.6–36.2)	31.7 (25.1–37.8)	5.3 (0.0–12.2)	17.1 (6.8–26.2)	26.4 (16.3–35.3)	36.1 (26.1–44.8)
China (rural)	3.4 (1.7–5.1)	5.9 (0.5–11.0)	25.7 (14.5–35.3)	No association	4.3 (0.0–10.5)	7.7 (1.4–13.6)	33.8 (18.9–46.0)
India (urban)	5.4 (0.1–10.4)	6.6 (0.0–17.1)	13.8 (4.9–21.9)	19.7 (11.7–27.0)	30.0 (18.0–40.3)	25.1 (9.6–37.9)	25.3 (10.4–37.7)
Weighted mean	10.5	20.9	25.1	19.6	28.3	33.4	38.3

In Table 5, the independent associations between individual frailty indicators (from both frailty paradigms) and incident dependence and mortality are presented. Data from all sites were combined together and meta-analysed to estimate pooled effect sizes. After controlling for demographic factors and chronic health conditions, weight loss, underactivity, slow walking speed, and cognitive impairment were associated with both outcomes. Undernutrition (arm circumference) was particularly strongly associated with mortality, but was not associated with incident dependence. Conversely, sensory impairment was weakly associated with onset of dependence, and was not associated with mortality. Exhaustion was not associated with either outcome. Heterogeneity in the effect sizes among sites was negligible to moderate, and only statistically significant for the associations between slow walking speed and cognitive impairment with incident dependence, and for the association of weight loss with mortality.

**Table 5:** Meta-analysed pooled effect sizes for the independent associations between individual frailty indicators and incident dependence and mortality

	Associations with incident dependence		Associations with mortality	
Frailty indicator	Mutually adjusted^a^ pooled effect size (IRR) ^b^	Test for heterogeneity	Mutually adjusted^a^ pooled effect size (HR)^c^	Test for heterogeneity
Exhaustion	F^d^ = 1.03 (0.90–1.17)	Cochrane’s Q 8.6, 8 df, *P* = 0.37	F = 1.00 (0.90–1.12)	Cochrane’s Q 10.4, 9 df, *P* = 0.32
		Higgins I^2^ = 7 (0–67)		Higgins I^2^ 13 (0–55)
Weight loss	F = 1.31 (1.06–1.61)	Cochrane’s Q 8.4, 6 df, *P* = 0.21	F = 1.40 (1.19–1.64)	Cochrane’s Q 17.7, 9 df, *P* = 0.04
		Higgins I^2^ = 28 (0–69)	R^5^ = 1.45 (1.13–1.87)	Higgins I^2^ 49 (0–75)
Underactivity	F = 1.35 (1.10–1.67)	Cochrane’s Q 11.8, 8 df, *P* = 0.16	F = 1.53 (1.32–1.88)	Cochrane’s Q 12.8, 9 df, *P* = 0.17
		Higgins I^2^ = 32 (0–69)		Higgins I^2^ 30 (0–66)
Slow walking speed	F = 1.28 (1.12–1.47)	Cochrane’s Q 17.0, 8 df, *P* = 0.03	F = 1.36 (1.21–1.51)	Cochrane’s Q 14.7, 9 df, *P* = 0.10
	R = 1.30 (1.05–1.61)	Higgins I^2^ = 53 (0–78)		Higgins I^2^ 39 (0–71)
Sensory impairment	F = 1.14 (1.01–1.29)	Cochrane’s Q 7.2, 8 df, *P* = 0.52	F = 1.03 (0.93–1.14)	Cochrane’s Q 6.9, 9 df, *P* = 0.65
		Higgins I^2^ = 0 (0–65)		Higgins I^2^ 0 (0–62)
Cognitive impairment	F = 1.53 (1.30–1.79)	Cochrane’s Q 16.9, 8 df, *P* = 0.03	F = 1.38 (1.23–1.54)	Cochrane’s Q 15.1, 9 df, *P* = 0.09
	R = 1.48 (1.16–1.90)	Higgins I^2^ = 53 (0–78)		Higgins I^2^ 40 (0–71)
Undernutrition (arm circumference <22 cm)	F = 1.11 (0.89–1.38)	Cochrane’s Q 10.4, 6 df, *P* = 0.11	F = 1.72 (1.47–2.01)	Cochrane’s Q 9.2, 9 df, *P* = 0.41
		Higgins I^2^ = 42 (0–76)		3 (0–63)

^a^The effect of each frailty indicator is adjusted for all of the others, in models also controlling for age group, sex, level of education, and health conditions (10/66 or DSM-IV dementia diagnosis, ICD-10 depression, number of physical impairments and stroke); ^b^IRR, Incidence rate ratio; ^c^HR, Hazard ratio; ^d^F, Pooled fixed effect; ^e^R, Pooled random effect (estimated only in the presence of statistically significant heterogeneity)

## Discussion

We have found, in a large population-based cohort study in LMICs, that both the physical and multidimensional frailty phenotypes predict the onset of dependence and mortality, even after adjusting for chronic diseases and baseline disability scores (Table 2). However, analysis of PAFs suggests that treating the number of underlying frailty indicators as ordinal scales, and, to an even greater extent, considering the aggregate effect of individual frailty indicators, provides a better overall prediction of risk of experiencing these adverse outcomes. Combining the seven indicators underlying both phenotypes provided the best overall prediction. While some of these seven indicators (weight loss, under activity, slow walking speed, and cognitive impairment) predicted both dependence and mortality, undernutrition predicted mortality only, and sensory impairment predicted dependence only; self-reported exhaustion predicted neither outcome.

The analyses were conducted on large population-based samples in Latin America, India, and China, hence allowing us to assess the consistency or cultural specificity of the observed associations. The study design was prospective, limiting information bias, with modest attrition. Measurement error will have occurred, but, if random, the effect will have been systematically to underestimate the effect of frailty exposures on mortality and dependence. We studied a wide range of frailty indicators comprising most of those included in the most widely used frailty phenotypes. Walking speed, undernutrition and cognitive impairment were measured objectively, an advantage over some other studies that relied entirely on self-report [16]. Visual and auditory impairment probably would also have been more accurately and appropriately assessed by objective testing. We were able to control fairly comprehensively for physical, mental, and cognitive disorders that are the major predictors of mortality and dependence, and for disability, hence precisely estimating the independent contribution of frailty to those outcomes. Handgrip strength was not measured in our surveys; thus, our physical frailty construct is only an approximation to the original Fried definition. The impact of this omission is difficult to assess. A recent meta-analysis indicates that handgrip strength is a consistent predictor of mortality, although effect sizes vary markedly among studies [31]. While, in this meta-analysis, the effect sizes for walking speed were larger, the authors cautioned against inferring too much from this finding, pointing out the few studies of walking speed, the correlation among frailty indicators, and the few studies that had estimated their independent effects. In two longitudinal studies that did seek to do this, the effect of handgrip strength on incident disability [2, 32] and mortality [32] were attenuated and no longer significant when adjusted for other frailty indicators and potential confounders [2, 32]. We also acknowledge that the only definition of self-reported weight loss available in our study (>4.5 kg in the last three months) is more precipitate than that used in the Fried criteria (>4.5 kg in the past year), and may inflate the association between that criterion and mortality due to the marked weight loss associated with terminal conditions.

Our findings regarding the predictive validity of the two frailty models are partly consistent with other studies. In a 4-year prospective community-based cohort study in three French cities, the Fried frailty phenotype was associated with in increased incidence of disability, independent of cognitive impairment [15, 33]. In the 12-country Survey of Health and Retirement in Europe, those meeting Fried frailty criteria had nearly a five times higher odds of death compared to non-frail individuals [34]. Most frailty studies have been conducted in developed countries. Two prospective studies from China and one from Mexico have demonstrated prospective associations between frailty indices (a composite of indicators of physical impairment, chronic disease diagnoses, activity limitation and disability) and mortality [35–37]. The justification for considering frailty as a unitary construct (‘frailty’ rather than ‘frailties’) seems not previously to have been subjected to critical empirical examination. Our finding that slow gait speed, low physical inactivity, weight loss, and cognitive impairment were associated both with mortality and dependence, but that self-reported exhaustion was associated with neither outcome, replicates precisely a finding from an earlier North American cohort study [32]. Variable predictive associations among frailty indicators explain our finding that the overall prediction of mortality and dependence is significantly reduced when the information from the various indicators is summarised as a dichotomous syndrome. The implicit assumption, that these are all indicators of a unidimensional latent trait, is challenged by our finding that the prediction provided by the aggregate of individual indicators exceeds that when the indicators are summed to form an ordinal scale.

## Conclusions

The results of our study support an emerging consensus that further empirical work on the scope and dimensionality of frailty, and the construct validity of its assessments should be a priority for future research [38]. However, even at this early stage in the detailed conceptualisation and measurement of frailty, it seems clear that information regarding variation in patterns of age-related change in physiologic and organ/system function can help to stratify risk for dependence and death, over and above any prediction provided by clinical diagnoses and disability. This principle extends to LMIC settings, according to the findings reported in this paper. Frailty is a key outcome in monitoring the public health response to the challenges of global population ageing, in particular the holy grail of compression of morbidity. Frailty indicators may assist in developing and targeting effective primary and secondary prevention strategies to delay or prevent the onset of dependence, and in providing holistic, coordinated care for older people with complex multimorbidities, particularly at the primary care level [39]. Evidence presented here supports the view that frailty is likely to be a multidimensional construct [38], and that we need therefore to consider ‘frailties’ in different organ-based and physiological systems, and their individual and joint impacts on functional decline, loss of independence and survival. There are likely to be benefits in moving beyond the physical frailty phenotype to consider at least the effects of chronic undernutrition and sensory and cognitive impairment [38]. A broader range of frailty indicators may cluster into meaningful sub-domains of frailty with common underlying pathophysiological mechanisms [40]. It is likely that more objective measurement of frailty indicators (including underlying physiological biomarkers) may provide better risk stratification. A better understanding of the frailty phenotypes and the pathways to adverse outcomes could inform simple multi-dimensional assessment and multi-component intervention strategies with considerable potential to add life to years as well as years to life [41]. Such approaches may have particular value in resource-poor LMIC settings, where population ageing is proceeding most rapidly, dependence is already highly prevalent [25], and where numbers of dependent older people are forecast to quadruple between 2000 and 2050 [41].

## Abbreviations

10/66 DRG: Dementia Research Group
IRR: Incident rate ratio
LMIC: Low- and middle-income country
PAF: Population attributable fractions
